# Richness and Cover of Nontimber Economic Plants along Altitude in Temperate Himalayan Forest-Use Types

**DOI:** 10.1155/2014/748490

**Published:** 2014-06-16

**Authors:** Akash Tariq, Muhammad Adnan, Naser M. AbdElsalam, Hassan Fouad, Kamran Hussain, Riaz Ullah, Ahsan Ullah

**Affiliations:** ^1^Department of Botany, Kohat University of Science and Technology, Kohat 26000, Pakistan; ^2^Riyadh Community College, King Saud University, Riyadh 11437, Saudi Arabia; ^3^Biomedical Engineering Department, Faculty of Engineering, Helwan University, Helwan 11792, Egypt; ^4^World Wide Fund for Nature, Peshawar 25000, Pakistan; ^5^Department of Chemistry, Government College Ara Khel, FR Kohat, Khyber Pakhtunkhwa 26000, Pakistan

## Abstract

Pakistani Himalaya stretches over a wide range of altitudinal gradients and supports high diversity of medicinal plants that are an important source for rural livelihood. Altitudinal effects on ground vegetation have already been indicated but ground vegetation is also under severe threat of grazing and over collection. The present study investigated the effect of altitude on medicinal plants abundance in both old-growth and derived woodland forests. Each of the five line transects was selected in old-growth and derived woodland forests. Each line transect consisted of four plots distributed at four altitudinal levels (2200, 2300, 2400, and 2500 m asl). Species richness under derived woodland had shown strong negative correlation (*r* = −0.95) with altitude while it was found to be nonsignificant under old-growth. Cover of most of the species such as* Veronica laxa* (*r* = −0.95, *P* ≤ 0.05) had shown significant negative correlation with altitude under derived woodland. Cover abundance of some species like* Valeriana jatamansi* and Viola* canescens* has also shown significant negative correlation under old-growth forest. Derived woodland can decrease the cover abundance of valuable medicinal plants towards extension at higher altitudes. Thus, protection of the derived woodland could serve as a tool for the improvement of rural livelihood and ecological restoration.

## 1. Introduction

Medicinal plants are an important component of NTFPs with a global market value of 83 million US dollars [1, 2]. These plants are playing a vital role in providing primary healthcare facilities and cash income to population throughout the world, particularly in developing countries [3–5]. Since early 1990s the role of NTFPs in sustainable forest-use and poverty alleviation has received considerable attention [6].

Pakistan is bestowed with diversity of medicinal plant resources that are used by marginal communities for domestic and commercial purposes [7]. The flora of the country is mostly confined to the Hindu Kush, Himalaya, and Karakorum regions [8, 9]. The inhabitants of Himalayan region have rich knowledge of using medicinal plants for traditional purposes [10, 11]. These plants not only are used for the treatment of fever, chest, and intestinal diseases as healing agents but also serve as an important raw material for manufacturing of traditional and modern medicines [7]. As one of the major NTFPs, local people collect approximately 600 species of medicinal plants [12], of which 500 are generally used in traditional health care practices and 350 are traded for millions of US dollars in the national and international markets [13]. About sixty thousand traditional practitioners (hakeems) in rural and remote areas make use of more than 200 plants as household remedies for curing several diseases [14].

Altitudinal gradient is one of the decisive factors having linear relationship with species richness [15] and species cover [16]. Besides climatic and other abiotic factors, altitudinal patterns of human use of forests can also exert influence on altitudinal species distributions. In the mountainous areas, low altitude regions are affected by settlements resulting in exploitation of forest resources. On the other hand high altitude regions are subjected to grazing intended to maintain grassland, with most forests remaining at midaltitude [17]. Heavy grazing is known to cause simplification of ecosystem structure, life forms, and species diversity, a situation which may eventually lead to impaired ecosystem functioning [17, 18].

This study was conducted in Nathiagali and its surrounding forests situated in North-West Pakistan, where local people use forests intensively for grazing and collection of medicinal plants. According to Nogués-Bravo et al. [19], most of the studies have been conducted on plants diversity and abundance along altitudinal gradient in disturbed forests. Nevertheless species diversity has been higher in the middle altitude (2100–2500 m asl) compared to lower (1400–1800 m asl) and higher altitudes (2800–3200 m asl) [20, 21]. Few published papers, however, reported on studies related to altitudinal effects on nontimber economic plants in Pakistan. This study was designed to find out variation in medicinal plants richness and cover abundance along altitude in two different forest-use types.

## 2. Methodology

### 2.1. Study Area

**Figure 1:**
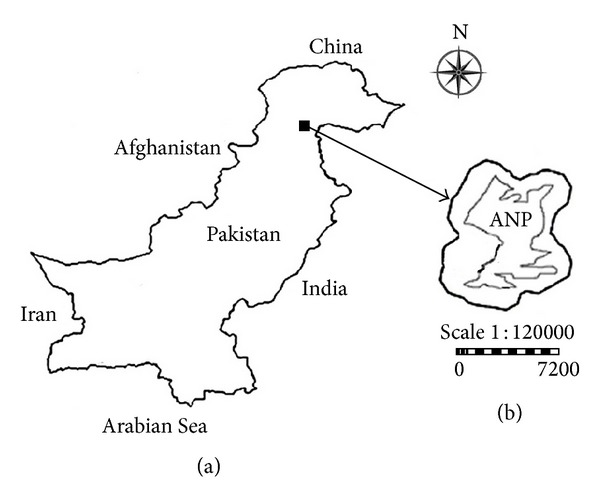
Map of the study area. (a) Pakistan and the location of the study region. (b) The study region with the Ayubia National Park (ANP) boundary (inside boundary) and its surrounding forests (outside boundary).

The study was carried out in Nathiagali located in North-West Pakistan (Figure 1(a)). It lies between 34°-01 and 34°-38 latitude and 73° and 22.8 E longitudes in the moist temperate Himalayan zone. The altitude varies between 1220 m and 2865 m asl. The study area included Ayubia National Park (ANP) having an area of 3312 ha (Figure 1(b)). There are 12 villages in the periphery of ANP with a population of about 50,000 people living in 8000 households. The community living around the park is dependent on its resources for fodder, livestock grazing, fuelwood, timber, and NTFPs [22].

### 2.2. Forest-Use Types

For this study, two forest-use types were differentiated on the basis of forest structure, management, land tenure, and resource utilization and were classified according to the nomenclature proposed by Putz and Redford [23].

Old-growth forest refers to the forest-use type with little or no human disturbance. It consists of many large diameter trees and the forest canopy is mainly closed. Old-growth forest can mainly be found in the ANP and in its surrounding reserve forests and should consequently be afforded the highest level of protection from resource exploitation.

Derived woodland refers to forest under high grazing pressure and where local inhabitants frequently collect fodder and medicinal plants. This forest-use type is generally adjacent to villages and contributes a major portion to the livestock fodder consumption. Legal and illegal logging have been taking place in the recent past.

### 2.3. Sampling Design and Plot Selection

**Figure 2:**
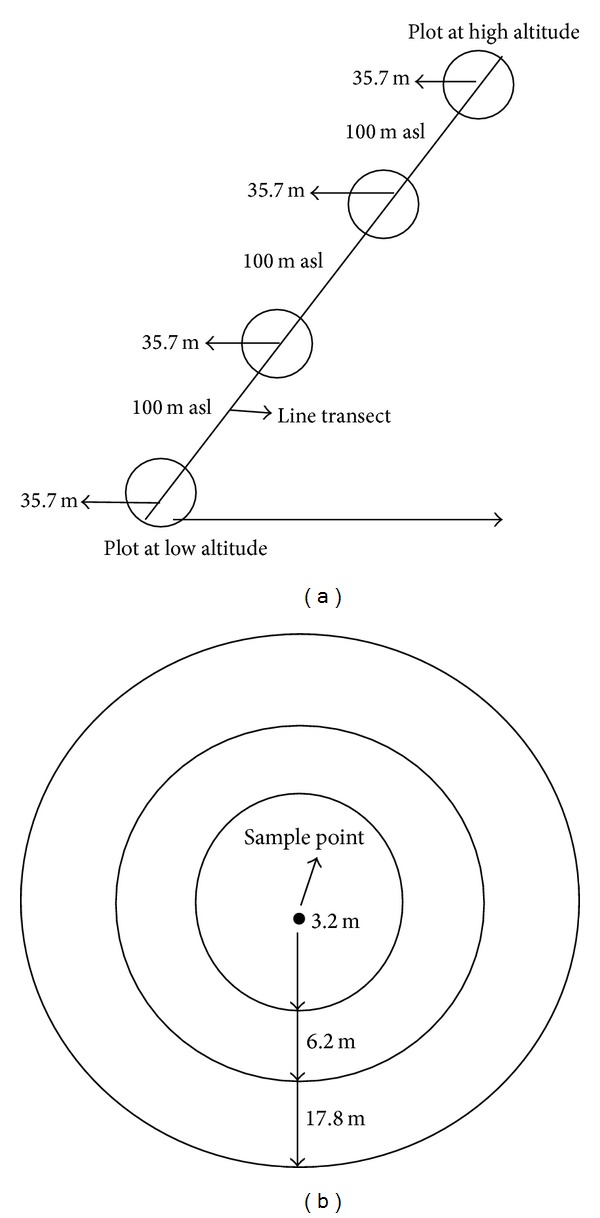
Line-transect and plot design. (a) represents line-transect including the plots starting from low altitude to higher altitude. (b) represents each single plot and sub-plots within it. This figure is a modified form of the line-transect method [24]. asl shows above the sea level.

Five line transects were laid in each forest-use type directed from low to high altitude [24]. The starting point of each line transect was a random sample point at 2200 m asl in Northern aspect. The sample points were located in the field with the help of global positioning system (GPS) [16]. Four circular plots were laid down at four different altitudes (2200, 2300, 2400, and 2500 m asl) on each line transect and the distance between every two plots was 100 m asl (Figure 2(a)). Slope correction was made accordingly for each plot. This altitudinal range is the middle altitudinal range of the study area where there is heavy grazing pressure and anthropogenic activities also take place up to some extent. Each circular plot had a diameter of 35.7 m, which was further divided into three parts. The plot with the radius of 17.85 m (1000 m^2^ area) was used for tree inventory. The middle part of the plot with radius of 6.2 m (120 m^2^ area) was used for shrub survey and the inner smaller part with the radius of 3.2 m (40 m^2^ area) was used for herb survey [25] (Figure 2(b)).

### 2.4. Nontimber Economic Plants Assessment

Studied variables were medicinal plant's species richness, frequency, and cover. Data on percentage cover of each medicinal plant species was collected by visual estimation method. Species richness was derived by counting the number of species in a plot. Percentage frequency was calculated by estimating the absence and presence of species in plots [26]. Assessment of each variable was carried out by taking average of five plots in five transects lying on the same altitude of given forest-use types.

Data collection was carried out from July to September, 2012. Information on ethnomedicinal uses and other nontimber forest product's uses were collected for those herb/fern species that were encountered in the ecological survey. Scientific names, family names, and publication authors were corrected according to the flora of Pakistan and the software Index Kewensis version 2.0 [27]. Related information such as local names, part use, uses as NTFPs, and medicinal uses were collected by interviewing local residents and herbalists of Nathiagali through questionnaire survey. Most local names of plants were in local language “*Hindko*.” A plant may have been used for only one part or several parts such as leaves, whole plant, roots or tuber, stem or bark, flowers, and seeds or fruits. Species used as NTFPs were placed under 8 categories: medicinal, fodder, ethnoveterinary, vegetables, insecticides, fruit, handicraft, and dye. Medicinal uses were divided into 28 categories (Table 1). Data on most of the species for their adaptation to sun or shade was collected from online web database Plants For A Future [7]. However, some of the species were not listed in the database; therefore, information on those species was collected from the local source. They were divided into five groups in terms of adaptation to various light conditions such as deeply shaded species, deeply shaded to partially shaded species, partially shaded species, partially shaded to sunny species, and sunny species.

### 2.5. Statistical Analysis

Spearman's correlation was performed between the altitude with medicinal plants cover and richness in a given forest-use type. Mann-Whitney* U* test was applied at the species level on individual medicinal plants cover, while as a whole on the species richness at any two similar altitudes between the two forest-use types. Data compilation, Spearman's correlation, and Mann-Whitney* U *test were carried out using Microsoft Excel and SPSS version 16.0 [28].

## 3. Results

### 3.1. Nontimber Economic Plants and Their Uses

**Figure 3:**
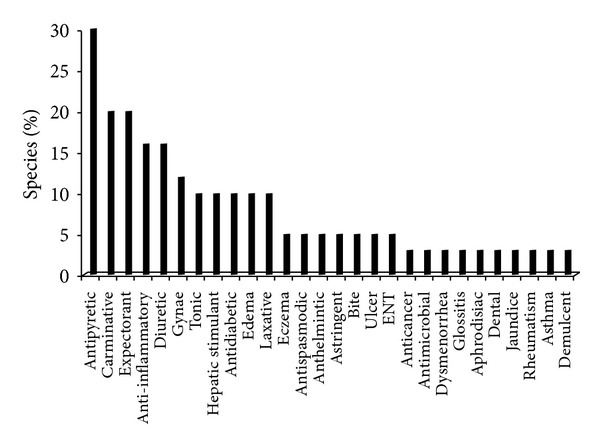
Percentage of medicinal plant species used for curing various diseases.

In total 31 medicinal plants belonging to 23 families were recorded in the two forest-use types. Thirty plant species were observed in derived woodland in comparison to 22 species in the old-growth forest. Twenty-one species were common in both forest-use types. Majority of the plants were adapted to the condition from partially shaded to sunny (40%) followed by deeply shaded to partially shaded (20%). Mostly leaves of the species (43%) were used as NTFPs particularly for medicinal purposes. Majority of plants (80%) were used medicinally followed by 60% as fodder species (Table 1). About 30% of the medicinal plant species were used as antipyretic followed by 20% as expectorant (Figure 3). From the market point of view,* Adiantum incisum* (8.1 US dollars Kg^−1^),* Fragaria nubicola *(8.1 US dollars Kg^−1^),* Dryopteris ramosa *(7.1 US dollars Kg^−1^),* Veronica laxa* (4.0 US dollars Kg^−1^), and* Viola canescens* (3.1 US dollars Kg^−1^) were found to be the most valuable species economically (Table 1).

### 3.2. Species Richness and Frequency

**Figure 4:**
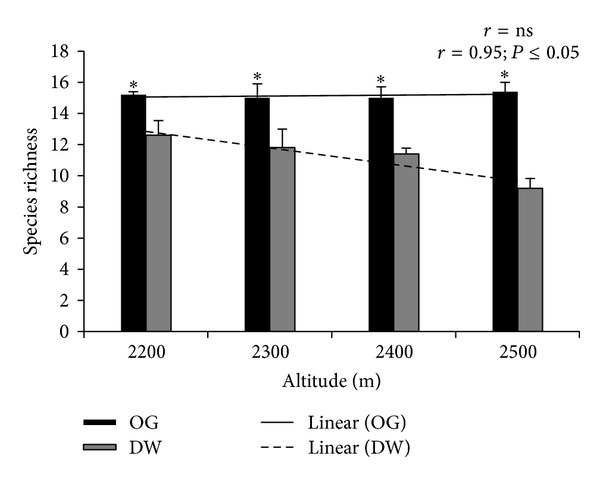
Species richness in old-growth (OG) forest and derived woodland (DW) along altitude. “*” represents significant difference between the two forest-use types. “*r*” represents Spearman correlation. ns indicates no significant correlation.

Plot level species richness at 2500 m asl was significantly higher (*P* ≤ 0.05) under old-growth forest (15 species) compared to derived woodland (9 species). The species richness showed negative correlation (*r* = 0.95; *P* ≤ 0.05) with altitude under derived woodland; however, it was not different in old-growth forest (Figure 4).

Nine species in old-growth forest like* Adiantum incisum*,* Dryopteris ramosa,* and* Fragaria nubicola* were more frequent showing 100% frequency almost at all altitudes. In derived woodland forest, five species* Dryopteris ramosa*,* Fragaria nubicola*,* Aquilegia pubiflora, Adiantum incisum, *and* Lygodium jopanicum* showed almost 100% frequencies (Table 2).* Veronica laxa* showed decrease in frequency with altitude in derived woodlands and was absent at the highest altitude surveyed (Table 2).

### 3.3. Species Cover

**Figure 5:**
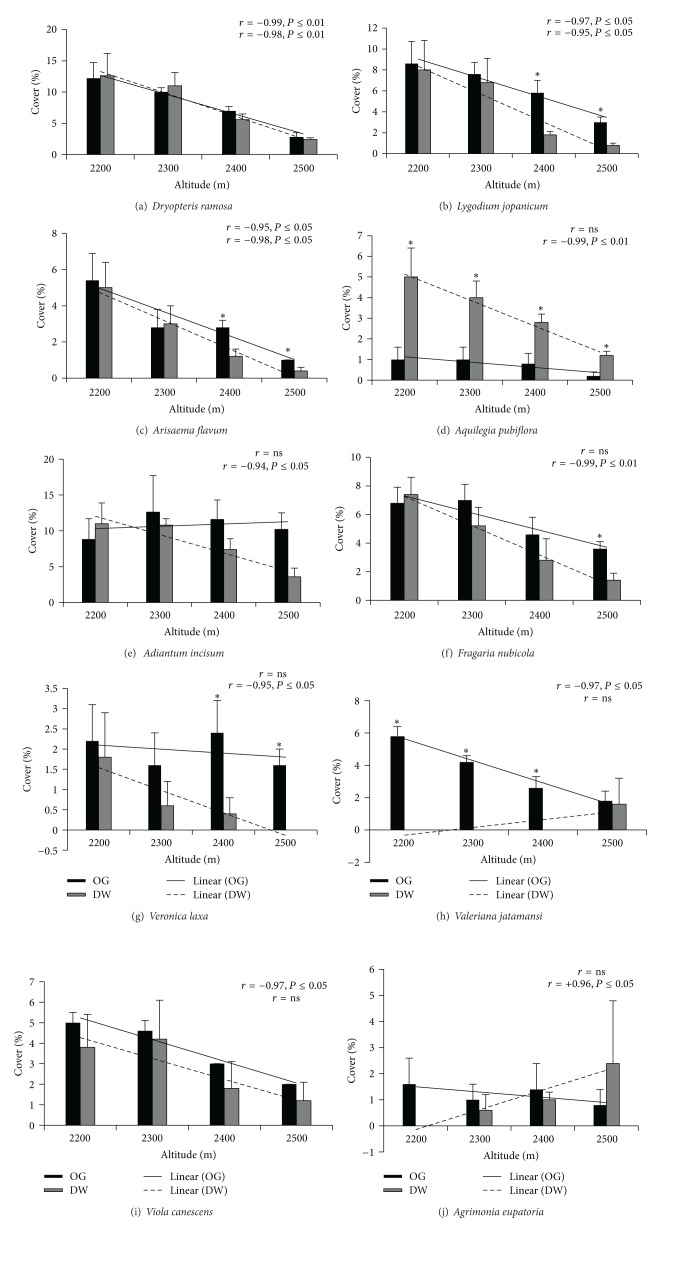
Cover of individual medicinal plant species in old-growth (OG) forest and derived woodland (DW) along altitude. * represents significant difference between the two forest-use types. *r* represents Spearman correlation. ns indicates no significant correlation.

Individual species had showed different patterns in cover abundance along altitude between the two forest-use types. Species cover of* Dryopteris ramosa* showed significantly negative correlation with altitudinal gradient in both old-growth forest (*r* = −0.99; *P* ≤ 0.01) and derived woodland (*r* = −0.98; *P* ≤ 0.01) (Figure 5(a)).* Lygodium jopanicum* and* Arisaema flavum* also showed similar trends (Figures 5(b) and 5(c)). Cover of* Fragaria nubicola *was negatively correlated (*r* = −0.99; *P* ≤ 0.01) with altitude under derived woodland forest while it was found indifferent under old-growth forest (Figure 5(f)).* Adiantum incisum, Aquilegia pubiflora, *and* Veronica laxa* showed similar trends (Figures 5(d), 5(e), and 5(g)).* Agrimonia eupatoria* was the only species which showed significant positive correlation (*r* = +0.96; *P* ≤ 0.05) with altitude in derived woodland (Figure 5(j)). Moreover, cover of* Aquilegia pubiflora* under derived woodland forest was significantly higher than its cover in old-growth forest at all altitudes (Figure 5(d)).* Valeriana jatamansi* cover was negatively correlated (*r* = 0.97; *P* ≤ 0.05) with altitude in old-growth forest, while it was not different and had limited occurrence under derived woodland (Figure 5(h)). A similar pattern was observed for* Viola canescens *(Figure 5(i)).

## 4. Discussion

Nontimber forest products in general and medicinal plants in particular are an essential source of income for the local people of Himalayan region of Pakistan [12]. The present study provided information on the ethnomedicinal uses of herbs species and their occurrence and abundance patterns along altitudinal gradient. The study indicated that local community of Nathiagali possesses valuable traditional knowledge on the uses of medicinal plants. Indigenous ethnobotanical knowledge at ANP has been transferred orally from generation to generation maintaining a strong interrelationship between people and plants [29]. The local residents living in these mountainous areas use indigenous knowledge to treat a variety of diseases [30, 31]. They know the preparation of raw drugs from herbs through personal skills and ancestral prescription [32]. These drugs were regularly used and have proven to be effective, cheap, and beneficial with almost no side effects compared to the allopathic drugs that are beyond the reach of the poor [29, 31]. The study showed that most of plant species reported from the region were used as antipyretic and expectorant. This might have been caused due to bad hygienic conditions and fuelwood smoke [7]. These findings are similar to an ethnobotanical study conducted in the Naran Valley of Pakistan, where species are locally used for such medicinal purposes [33].

Medicinal plants market in Pakistan is growing at an annual rate of 15% [12]. Medicinal plants marketing plays an important role in the socioeconomic conditions of its dependent people by contributing to their total annual income [7, 34]. Species such as* Dryopteris ramosa, Fragaria nubicola, Veronica laxa, Valeriana jatamansi,* and* Viola canescens *are highly traded medicinal plant species in the region. However, such plant resources are under severe threat of grazing and overcollection particularly at the higher altitudes of the region [16].

In derived woodland, medicinal plant specie's richness decreased as altitude increased, which might be due to grazing pressure coupled with other anthropogenic activities [16]. The total number of species recorded was higher in derived woodland compared to old-growth forest. Possible reason could be relatively opened canopy in derived woodland, which provided congenial environment for the growth of species that were more adapted to sunny conditions [35]. Canopy cover can have considerable effects on plant species diversity due to altering the light conditions for the understory species [36]. In addition, in forests having no or little disturbance, only the competitive dominants can survive, while at sufficient high level of disturbance only fugitive species can survive [37, 38]. On the other hand, the study pointed out that species richness at plot level was higher under old-growth forest compared to derived woodland. This could be because of more species similarity under old-growth forest compared to derived woodland [39]. Moreover, in old-growth forest species richness did not show any significant decrease with altitude, which could be due to high level of protection under it [16].

Valuable species such as* Viola canescens *and* Valeriana jatamansi* were observed most abundantly and frequently in old-growth forest when compared to derived woodland. Adnan et al. [7] also reported similar results. This is most probably due to species adaptation to deeply shaded conditions provided by high tree basal area and tree canopy cover in old-growth forest [16, 40]. Cover of these species along with* Dryopteris ramosa, Lygodium jopanicum, *and* Arisaema flavum* was observed to be decreased as altitude increased under this forest-use type. On the other hand,* Veronica laxa* showed a high frequency at higher altitudes, while* Aquilegia pubiflora* showed low frequency at higher altitude in old-growth forest. Differences in altitudinal patterns of species abundance may also be caused by factors such as slope, aspect, soil, tree canopy cover, and species adaptation to the amount of light [36].

In derived woodland, certain species such as* Dryopteris ramosa*,* Lygodium jopanicum*,* Arisaema flavum*,* Aquilegia pubiflora*,* Fragaria nubicola*,* Adiantum incisum,* and* Veronica laxa* showed a significant decrease in cover with increase in altitude. Moreover,* Bistorta amplexicaulis* and* Veronica laxa* showed a decrease in frequency as altitude increases. These species are locally used as fodder and are economically important from the local's perspective.* Veronica laxa* was completely absent at higher altitude, which might be due to the high grazing pressure and overcollection. Grazing pressures are always directed toward higher altitudes to avoid any damage to the agricultural crops [19]. The indirect effects of overgrazing include soil compaction and mechanical injuries to seedlings, which increase susceptibility of the soil to erosion and reduce fertility. This can significantly reduce the abundance of ground vegetation [41].* Agrimonia eupatoria* has shown an increase in cover along altitudinal gradient because this is not a fodder species and is adapted from partially shaded to sunny condition [42].

## 5. Conclusions

In conclusion, forest-use types play a major role in the variation of cover abundance and species richness along altitudinal gradient. Multiused herb species can be vulnerable at higher altitudes due to high grazing pressure directed upwards. Decrease in herbs vegetation cover at higher altitudes could also decrease soil binding capacity, which could in turn increase chances of flooding in monsoon season. ANP and its surrounding forests consisted of economically and medicinally valuable flora that needed to be conserved for the ecological restoration and livelihood of the local people. Studies addressing the effects of forest stand structural variables coupled with environmental variables along altitude on medicinal plants are needed to know more about the ecology of such species.

## Figures and Tables

**Table 1:** Medicinal plants of Nathiagali (herbs and ferns).

Botanical name	Local name	Family	Part used∗	NTFPs uses∗∗	Medicinal uses∗∗∗	Light∗∗∗∗	Local price in US dollars	Market price
*Adiantum incisum *Forsk.	Kakhpai/Sumbal	Adiantaceae	ii	a, c	14, 12, 6	PS-S	1.02	8.1
*Agrimonia eupatoria *L.	Kanachika	Rosaceae	i	h	na	PS-S	na	na
*Ajuga bracteosa *Benth.	Ratti booti	Lamiaceae	ii	a	28, 3, 13, 1, 24	PS-S	2.04	2.04
*Androsace rotundifolia *Y. Nasir	Gari boti	Primulaceae	ii	a	15	PS-S	na	na
*Aquilegia pubiflora *Wal. ex. Royle.	Phool	Ranunculaceae	iv	a, c	6, 27	PS-S	na	na
*Arisaema flavum *(Forssk) Schott.	Adbis	Araceae	iii, iv	a, b, e, g	15, 5	PS-S	na	na
*Bistorta amplexicaulis *D. Don	Masloon	Polygonaceae	i, iii	a, c	14, 29, 17, 12, 6, 4, 9	PS	0.2	1.02
*Chenopodium album *	Sarmay	Chenopodiaceae	i	d	17	na	na	na
*Chrysanthemum leucanthemum *Linn.	Chity Phool	Euphorbiaceae	ii	c, f, g	na	S	na	na
*Clematis grata *Wall.	Birla, Ghrazela	Ranunculaceae	ii	a, c	14	DS-PS	na	na
*Dipsacus inermis *Wall.	Tandi	Dispaceae	i	d	na	PS-S	na	na
*Dryopteris ramosa* (Hope) C. Chir	Pakha	Dryopteridaceae	i	a, c, d	1, 8, 17, 18, 7	DS	1.02	7.15
*Euphorbia wallichii *Hook. F.	Harvi	Euphorbiaceae	iv	a, c	8, 15,	S	na	na
*Fragaria nubicola *Lindl. ex Lacaita	Pinjakha	Rosaceae	i, v	a, c, d	23, 4, 10, 19, 20	PS-S	2.04	8.1
*Galium aparine* L.	Kochan	Rubiaceae	ii	a, c	8, 12, 4, 23, 13	PS-S	na	na
*Hedera nepalensis *K. Koch (Syn: *H. helix* L.)	Albumber	Araliaceae	i, v	a, b, c	8, 12, 16	DS	0.1	5.11
*Impatiens bicolor *Royle	Batmandar	Balsaminaceae	v	a, c	1, 12, 23	DS-PS	na	na
*Leucanthemum vulgare *	Marguerite	Asteraceae	ii	c	na	S	na	na
*Lygodium jopanicum *L.	na	Lygodiaceae	na	na	12, 27	na	na	na
*Nepeta laevigata *(D. Don) Hand. -Mazz.	Muskbal	Lamiaceae	i	c	na	S	na	na
*Oxalis debilis* L.	Teenpatra	Oxalidaceae	na	na	22, 12, 26, 16, 21	na	na	na
*Polygonatum verticillatum *All	Krimcha	Liliaceae	i	a, c	6, 25, 2	DS-PS	2.04	8.1
*Prunella vulgaris *L.	na	Lamiaceae	na	na	4	na	na	na
*Ranunculus muricatus *L.	Ratmondia	Ranunculaceae	i	c	na	PS-S	na	na
*Rabdosia longituba *	Boi	Lamiaceae	iii	c	28	na	na	na
*Senecio salignus *DC.	Chita ula	Asteraceae	i, iii, v	a, b, c	17, 4, 5	DS	na	na
*Swertia chirata * Buch. -Ham. ex D. Don	Choreta	Gentianaceae	ii	a	14, 12, 21, 16	PS	0.5	4.08
*Urtica dioica *L.	Bichu boti	Urticaceae	i, iii	a, b, d	14, 12, 4, 22	PS-S	na	na
*Valeriana jatamansi *Wall.	Mushk-e-bala	Valerianaceae	i, iii	a, c	1, 8, 23, 10	DS-PS	0.1	1.02
*Veronica laxa *Benth.	Mashkanne	Plantaginaceae	i	a, c	14, 1	PS-S	0.5	4.08
*Viola canescens *Wall.	Swarphol/Banafsha	Violaceae	ii	a, c, d	8, 13, 22, 23, 9, 11	DS-PS	0.6	3.06

Part used∗: (i) leaves, (ii) whole plant, (iii) roots and tubers, (iv) stem, and (v) seeds and fruits. NTFPs uses∗∗: (a) medicinal, (b) ethnoveterinary uses, (c) fodder, (d) vegetables, (e) fruit, (f) handicraft, (g) insecticides, and (h) dye. Medicinal uses^∗∗∗:^ (1) tonic, (2) aphrodisiac, (3) dental, (4) anti-inflammatory, (5) bites (snakes, scorpion, etc.), (6) gynae, (7) anticancer, (8) carminative, (9) antispasmodic, (10) astringent, (11) demulcent, (12) antipyretic, (13) hepatic stimulant, (14) expectorant, (15) eczema, (16) antidiabetic, (17) laxative, (18) antimicrobial, (19) dysmenorrhea, (20) glossitis, (21) anthelmintic, (22) edema, (23) diuretic, (24) jaundice, (25) rheumatism, (26) asthma, (27) ulcer, and (28) ENT (ear, nose, and throat). Light requirement∗∗∗∗: (1) deeply shaded (DS), (2) partially shaded (PS), and sunny (S) [7]. na indicates not available.

**Table 2:** Frequency (%) of medicinal plant species.

Botanical names	Old-growth forest (%)				Derived woodland (%)			
	2200 asl *n* = 5	2300 asl *n* = 5	2400 asl *n* = 5	2500 asl *n* = 5	2200 asl *n* = 5	2300 asl *n* = 5	2400 asl *n* = 5	2500 asl *n* = 5
*A. flavum *	100	80	100	100	100	80	80	40
*A. incisum *	100	80	100	100	100	100	100	80
*A. pubiflora *	40	40	40	20	80	100	100	100
*A. rotundifolia *	na	na	na	na	0	0	20	0
*A. bracteosa *	na	na	na	na	40	40	20	40
*A. eupatoria *	40	40	40	40	0	20	80	20
*B. amplexicaulis *	80	100	100	100	60	20	20	20
*C. album *	40	20	20	20	20	20	0	0
*C. grata *	na	na	na	na	20	40	0	20
*C. leucanthemum *	20	40	40	60	20	0	20	0
*D. ramose *	100	100	100	100	100	100	100	100
*D. inermis *	20	40	0	0	0	20	20	20
*E. wallichii *	na	na	na	na	20	0	0	0
*F. nubicola *	100	100	100	100	100	80	80	80
*G. aparine *	na	na	na	na	0	20	0	0
*H. nepalensis *	60	80	80	80	0	20	20	0
*I. bicolor *	80	80	80	80	80	60	60	60
*L. jopanicum *	100	100	100	100	100	80	100	80
*L. vulgare *	na	na	na	na	20	20	0	0
*N. laevigata *	na	na	na	na	0	0	0	20
*O. debilis *	100	100	80	80	40	40	40	40
*P. verticillatum *	20	0	0	0	60	40	60	20
*P. vulgaris *	na	na	na	na	20	0	0	0
*R. longituba *	80	40	60	60	20	40	40	40
*R. muricatus *	100	100	100	100	60	60	80	40
*S. chirata *	na	na	na	na	40	40	40	40
*S. salignus *	20	40	40	20	na	na	na	na
*U. dioica *	60	60	60	40	0	0	20	20
*V. canescens *	100	100	100	100	60	60	40	40
*V. jatamansi *	100	100	80	80	0	0	0	20
*V. laxa *	60	60	80	100	40	20	10	0

“na” indicates not available and “asl” indicates above the sea level.
